# Seismic performance of soft soil foundation with a new type of assembled wall tuned mass damper

**DOI:** 10.1371/journal.pone.0298263

**Published:** 2024-05-09

**Authors:** Qi Jiang, Zhigang Yin, Hang Yin, Runbo Ma, Weiqiang Lin

**Affiliations:** 1 School of Mining, Liaoning Technical University, Fuxin, China; 2 Institute for Smart City of Chongqing University in Liyang, Liyang, China; 3 College of Civil Engineering, Liaoning Technical University, Fuxin, China; University of Zanjan, ISLAMIC REPUBLIC OF IRAN

## Abstract

The design of tuned mass damper (TMD) parameters is influenced by the soil-structure-TMD coupling system; thus, it is important to consider the soil-structure interaction (SSI) for the vibration control effect of the TMD. Recently, the acquisition of TMD parameters considering soil-structure interactions has only remained at the theoretical stage, lacking relevant experimental verification. Traditional TMD face the problems of occupying a large building space, increasing construction costs, and non-replaceable components. In this study, an assembled wall-type damping TMD was designed. By comparing the dynamic response of the uncontrolled and controlled structures equipped with the newly assembled wall-type damping TMD in the shaking table test on a soft soil foundation, we analyzed whether the SSI effect was considered in the TMD design parameters on the damping effect of the newly assembled wall-type tuned mass damper. The TMD parameters optimized using the artificial intelligence algorithm were verified experimentally. The results indicated that the traditional TMD design parameters were discordant because the SSI effect was not considered. The SSI effect in the soil effectively reduces the dynamic response of the superstructure. By considering the SSI effect and improving the multi-population genetic algorithm, a wall-type damping TMD with optimized parameters can achieve a good damping effect.

## 1 Introduction

With the continuous deepening of research on shock absorption technology and continuous changes in technology, structural vibration control technology has rapidly developed. Passive control has been widely used because it does not involve external forces. Among them, the Tuned Mass Damper (TMD), as a passive control facility, has the advantages of energy saving and a simple structure, and has become a hot spot of current application and development. However, in the existing research on TMD, structures and TMD systems are mostly based on rigid foundations [1–5], neglecting the impact of soil-structure interaction (SSI) on the performance of control systems. In practical engineering, structures are often built on foundations with different soil characteristics, and there is an undeniable interaction between the soil and upper structure. The presence of soil can change the frequency characteristics of the upper structure, and the sensitivity of the TMD parameters to frequency often results in no significant seismic reduction effect for TMD devices designed based on rigid foundation conditions [6–9]. Therefore, the SSI effect must be considered in the design of TMD with soft soil foundations.

Han et al. [10] established a vibration control equation for a fixed offshore wind turbine structure considering soil-structure interaction, revealing the time- and frequency-domain responses of the TMD damping mechanism and the impact of soil-structure interaction on vibration control effectiveness, demonstrating the importance of soil-structure interaction. Huang et al. [11] established a simplified computational model of a wind tower under seismic action, in which the soil-structure interaction was considered. The results showed that the greater the ground shaking intensity, the softer the site soil where the wind tower was located and the lower the seismic reduction efficiency of the TMD. Khoshnoudian et al. [12] studied the optimization design of a passive mass-tuned shock absorber system considering soil-structure interactions. The results indicated that the performance of the damper could be improved by considering the influence of soil–structure interactions. Farshidianfar and Soheili [13] used an ant colony optimization algorithm to optimize the design of high-rise structure TMD considering SSI effects and proved the effectiveness of ant colony optimization algorithms. Khatibinia et al. [14] used the Whale Optimization Algorithm (WOA) to optimize the structural TMD parameters considering the SSI effects, with the objective function set to minimize the structural response and TMD maximum displacement. The results indicated that the type of soil and setting of the objective function had a significant impact on the optimization design of the TMD system.

The above literature is based on numerical model calculations; however, there are relatively few shaking table experimental studies on TMD devices that consider the SSI effects. Lou et al. [15] conducted shaking-table tests on a soil-pile-structure TMD interaction system. The results showed that the soil-structure interaction effect reduced the vibration reduction efficiency of the TMD device, and in few cases, the TMD device amplified the dynamic response of the structure. Liu et al. [16] studied the vibration control performance of an eddy current-tuned mass damper (ECTMD) considering SSI effects on structures under earthquake action and conducted shaking table tests on a six-story steel frame model equipped with an ECTMD in the SSI system. They found that eddy current damping could improve the control performance of the TMD, especially in the SSI system, which had a significant improvement effect. However, this method is costly.

In addition, the design of traditional TMD is mostly a rigid connection between the TMD device and the controlled structure [17, 18], and the designed TMD is inconvenient for installation and disassembly, and the parameters cannot be flexibly adjusted, resulting in a mismatch phenomenon during the accumulation of structural damage, which affects the damping effect. Meanwhile, regardless of the type of TMD, its essence is to rely on large mass blocks to achieve tuning, which will inevitably increase the cost significantly and occupy limited space resources. Utilizing the quality of each component of the existing building to achieve the goal of shock absorption control is also a topic with broad research significance [19–22]. Therefore, the author designed a new wall-mounted shock absorber, TMD [23].

To test whether SSI effect is considered on the damping performance of new wall tuned mass dampers on soft soil, shaking table tests are conducted on both uncontrolled and controlled structures with and without SSI effect. For the wall-damping TMD device without the SSI effect, the TMD parameter calculation formula based on the rigid foundation deduction proposed by the authors [24] will continue to be adopted, whereas for the wall-damping TMD device considering the SSI effect, the improved multi-population genetic algorithm proposed by the authors [25] will be adopted to optimize its parameters. To compare and verify the damping performances of the two methods simultaneously, this study compares the damping performance of the uncontrolled structure under a rigid foundation in the literature [24] with that under a soft soil foundation to analyze the influence of the SSI effect on the structure. For specific parameters and implementation of the improved multi-population genetic algorithm, refer to the literature [24, 25].

## 2 Model soil and soil box production

### 2.1 Configuration and working mechanism of a new type of assembled wall tuned mass damper

**Fig 1:**
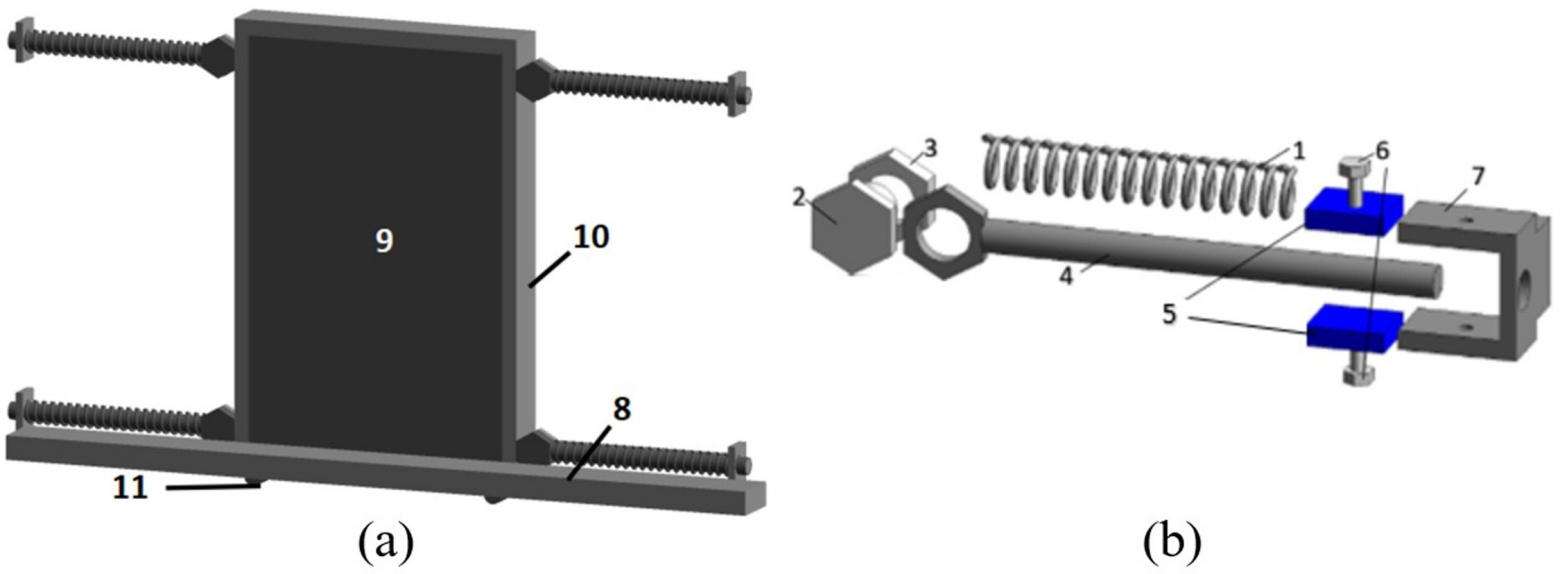
Wall damping TMD diagram: (a) Wall TMD model drawing; (b) Structure diagram of parameter adjusting rod.

The main feature of the wall-type TMD damping structure is the separation of the wall, which does not participate in the structural force in the traditional building structure, from the main load-bearing structure, and the two are connected to the compressive part through a damper (as shown in Fig 1). It consists of It consists of spring 1, high-strength bolt 2, nut 3, reset tie rod 4, friction rubber 5, pressure regulating bolt 6, embedded U-shaped steel sheet 7, baffle 8, mass block 9, iron frame 10 and roller group 11. In the overall structure, baffle 8 is connected to the steel sheet embedded in the structure by bolts, and the outer iron frame 10 of the wall is welded with nut 3 to form a mass block system together with the wall, and is fixed by high-strength bolt 2 together with reset tie rod 4. The end of the reset tie rod 4 was covered with friction rubber 5 and spring 1. When external excitation occurs, the wall TMD moves with the structure left and right under the action of inertial force. Spring 1 provides a restoring force for the structure, to ensure that the structure has a self-resetting function. Friction rubber 5 is connected to pressure regulating bolt 6, and the positive friction force between friction rubber 5 and reset tie rod 4 can be adjusted by twisting pressure regulating bolt 6 to provide the required damping for the structure. When assembling and disassembling, it is only necessary to twist the high-strength bolt 2 to separate the reset tie rod 4 from the mass block system and replace the structural accessories at will. The main structure comprises the vertical and horizontal stress components, and the wall-type TMD damping structure has the function of filling the building as well as organically combines with the energy dissipation and vibration reduction device as a mass block in the TMD system to form a TMD system to dissipate seismic energy. To prevent the TMD mass block from plane damage when excitation occurs, plane-limiting baffles are arranged on both sides of the isolation support; the specific structure can be found in [15].

### 2.2 Model similarity ratio design

In this study, based on the second (Buckingham) law of similarity, the similarity relationship of various physical quantities is determined by dimensional analysis, with the basic controllable similarity constants; the similarity relationship of other physical quantities can be calculated by dimensional analysis [26, 27]. According to the calculation results, the model structure is an under-artificial mass model. By adding an additional weight to the lead block [28, 29], the final model was similar, as listed in Table 1.

**Table 1:** Similar constant of shaking table test.

Physical Parameters	Relationship formula	Similarity constants (model/prototype)
Length 1	S_1_	0.1
Area S	${\text{S}}_{\text{S}}={\text{S}}_{\text{I}}^{2}$	0.01
Line displacement X	S_X_ = S_1_	0.1
Angular displacement β	S_σ_ = S_E_	0.35
Strain ε	S_ε_ = S_σ_/S_E_	1.0
Stress σ	S_σ_ = S_E_	0.35
Modulus of elasticity E	S_E_	0.35
Density ρ	S_ρ_ = Sσ/(S_a_⋅S_1_)	1.75
Quality m	${\text{S}}_{\text{m}}={\text{S}}_{\sigma }\cdot {\text{S}}_{1}^{2}/{\text{S}}_{\text{a}}$	1.75 ×10–3
Concentration F	${\text{S}}_{\text{F}}={\text{S}}_{\sigma }\cdot {\text{S}}_{1}^{2}$	3.5 × 10–3
Line load q	S_q_ = S_σ_∙S_1_	0.035
Surface load p	S_p_ = S_σ_	0.35
Bending moment M	${\text{S}}_{\text{M}}={\text{S}}_{\sigma }\cdot {\text{S}}_{1}^{3}$	3.5 × 10–4
Periodicity T	${\text{S}}_{\text{T}}={\text{S}}_{\text{l}}^{0.5}\cdot {\text{S}}_{\text{a}}^{-0.5}$	0.224
Frequency f	${\text{S}}_{\text{f}}={\text{S}}_{\text{l}}^{-0.5}\cdot {\text{S}}_{\text{a}}^{0.5}$	4.47
Stiffness	S_k_ = S_E_∙S_1_	0.035
Damping	${\text{S}}_{\text{c}}{\text{=S}}_{\sigma }{\times \text{S}}_{\text{1}}^{1.5}{\times \text{S}}_{\text{a}}^{-0.5}$	7.8 × 10–3
Speed v	S_v_ = (S_1_∙S_a_)^0.5^	0.447
Acceleration a	S_a_	2.0
Gravitational acceleration g	S_g_	1.0

### 2.3 Model soil box design

The shaking table test of soil-structure dynamic interactions usually requires placing the soil in a soil box, and the soil loses some of the parameter characteristics of the prototype soil before entering the laboratory. Owing to the limited size of the shaking table, this study selected a rigid model box that is widely used in domestic and foreign vibration equipment, and is characterized by a high total stiffness of the model box and small lateral deformation of the box wall during vibration. Owing to the high lateral deformation capacity of the box shape, the reflection of seismic waves at the boundary is strong. Therefore, in most tests, elastic materials must be pasted to weaken the boundary deformation of the soil and boundary effect of the model container.

**Fig 2:**
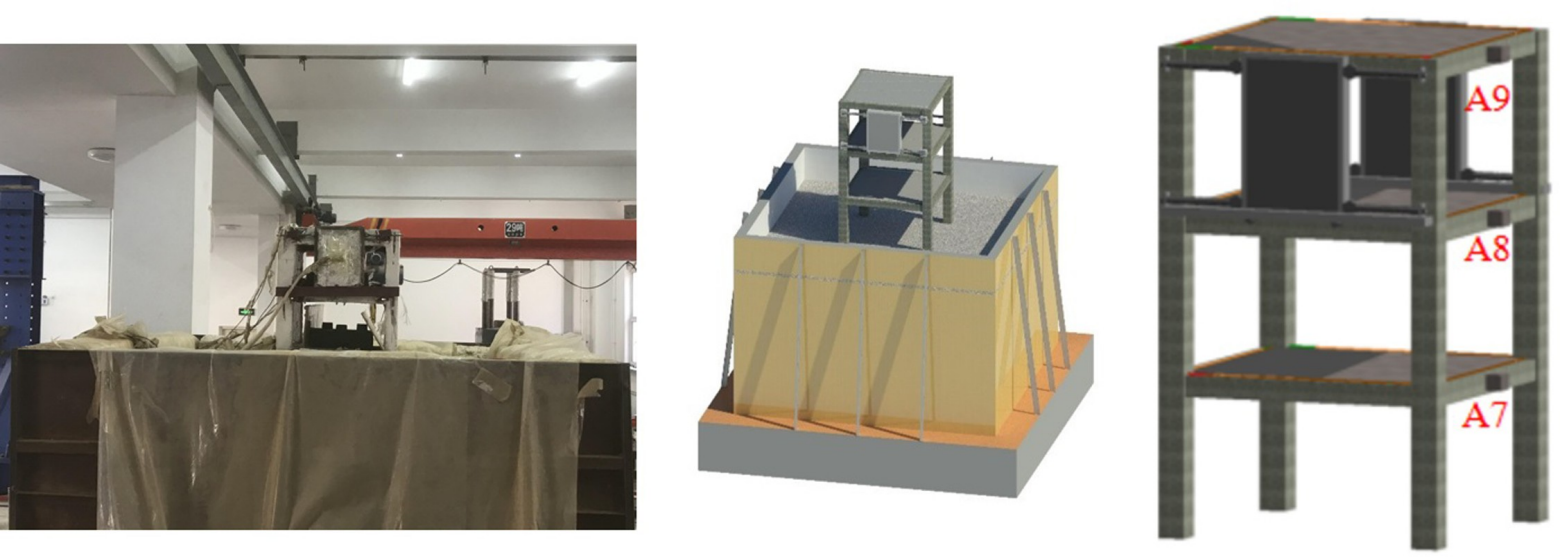
Model box structure.

Based on the above requirements, the soil box length × wide × high size set to 2.0 m ×2.0 m ×1.5 m in this study, 200 mm thick polystyrene foam and a layer of polyvinyl chloride film were attached to the inner wall to reduce the impact of boundary effect. Meanwhile, our research group also published relevant literature to prove the feasibility of a rigid model box with soft lining under this treatment method [30–32]. The box wall was fixed by welding square steel pipes and plates, and the bottom of the soil box was fixed to a shaking table using high-strength bolts. The final design molding effect is shown in Fig 2.

### 2.4 Model soil design

In the study of soil-structure shaking table tests, it was considered that excessive moisture content in the soil could damage the shaking and measurement equipment used in the test. Meanwhile, owing to the predominance of seismic phenomena in cohesionless soils, especially sand, there was a special sensitivity to various types of dynamic loading effects. Therefore, sand was used for experimental testing in this study.

Before the tests began, the sand was sent to the laboratory for testing. After drying, the impurities in the sand were removed, and the relative density was measured as 80%. According to the test, the elastic modulus of the soil was 13.69 MPa, and the Poisson’s ratio was 0.3. The shear wave velocity was obtained from the ratio of the distance between the accelerometer at the base of the model soil and the accelerometer on the surface of the model soil and the time difference of the collected waveform. The basic properties of the sand used in the final tests are listed in Table 2.

**Table 2:** Test sand performance indexes.

Density (g/cm^3^)	Relative density (%)	Poisson’s ratio	Internal friction angle (°)	Elastic modulus (MPa)	Shear wave velocity (m/s)
1.614	80	0.30	30	13.69	84

**Fig 3:**
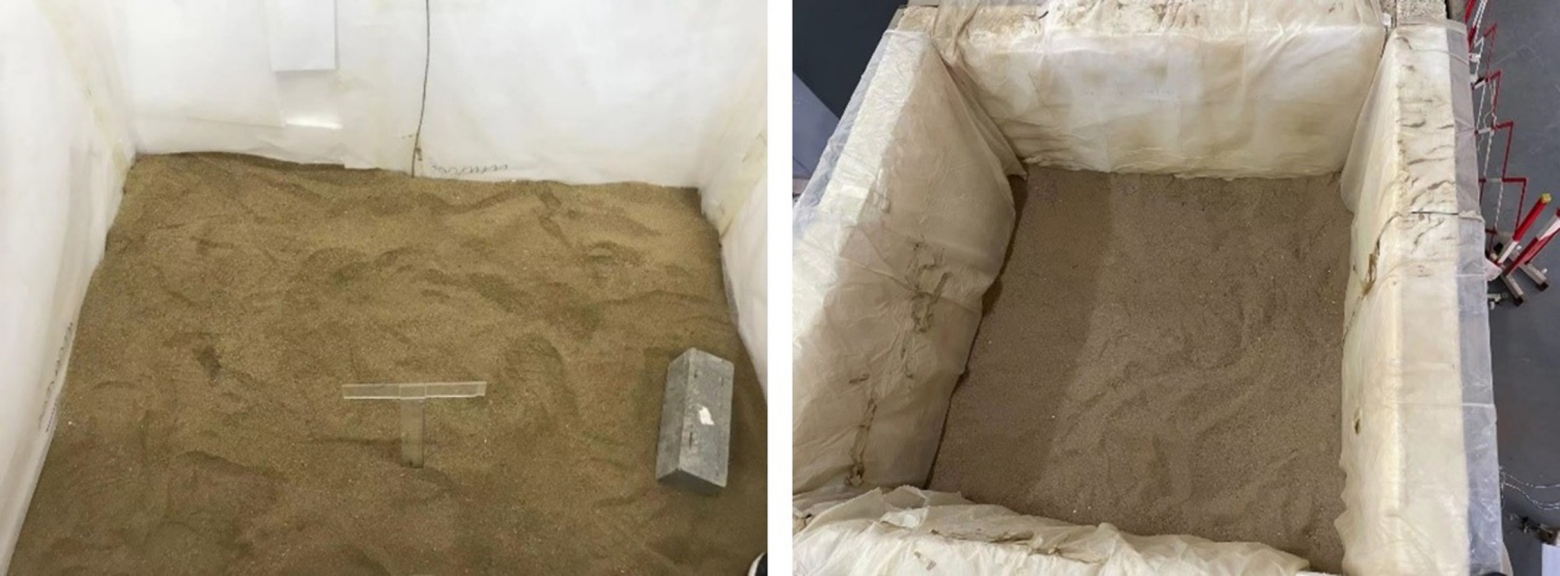
Test sand preparation.

Owing to the unique nature of the soil, it was difficult to achieve a consistent similarity relationship between the soil and structure in the model tests. When designing the experimental soil, the necessary similarity ratios can be controlled by adjusting the moisture content and dry density of the prototype soil to address the research problem. The model soil preparation process is as follows:

Large-sized sand and gravel that did not meet the test conditions were screened according to soil grading. To ensure the accuracy of the test data, it was necessary to control the moisture content strictly. After the moisture content met the requirements, the soil should be filled in five layers, with each layer height of approximately 0.25 m. Slowly spread the soil manually to evenly distribute the soil. After each layer was filled, the height and flatness of the soil layer should be checked, and the next layer should be continued to be filled. After lifting the model, the structure was allowed to stand for 24 h to ensure soil consolidation. Fig 3 illustrates the sand preparation process.

### 2.5 Layout of measurement points

In this test, a total of 12 acceleration sensors of type 1A202E (frequency response range: 0.2–1500 Hz, sensitivity: 101.3 mV/m/s^2^, range: ±5 g) and several strain gauge sensors were arranged to collect the acceleration of each layer of the model and measure the strain response of concrete and reinforcement. The acceleration sensors are represented by “A,” and the strain sensors are represented by “D.” A data acquisition system (DH5922, DH3817K) produced by China Donghua Measurement and Control Co., Ltd. was used to collect the acceleration and strain response data under seismic excitation for each case.

**Fig 4:**
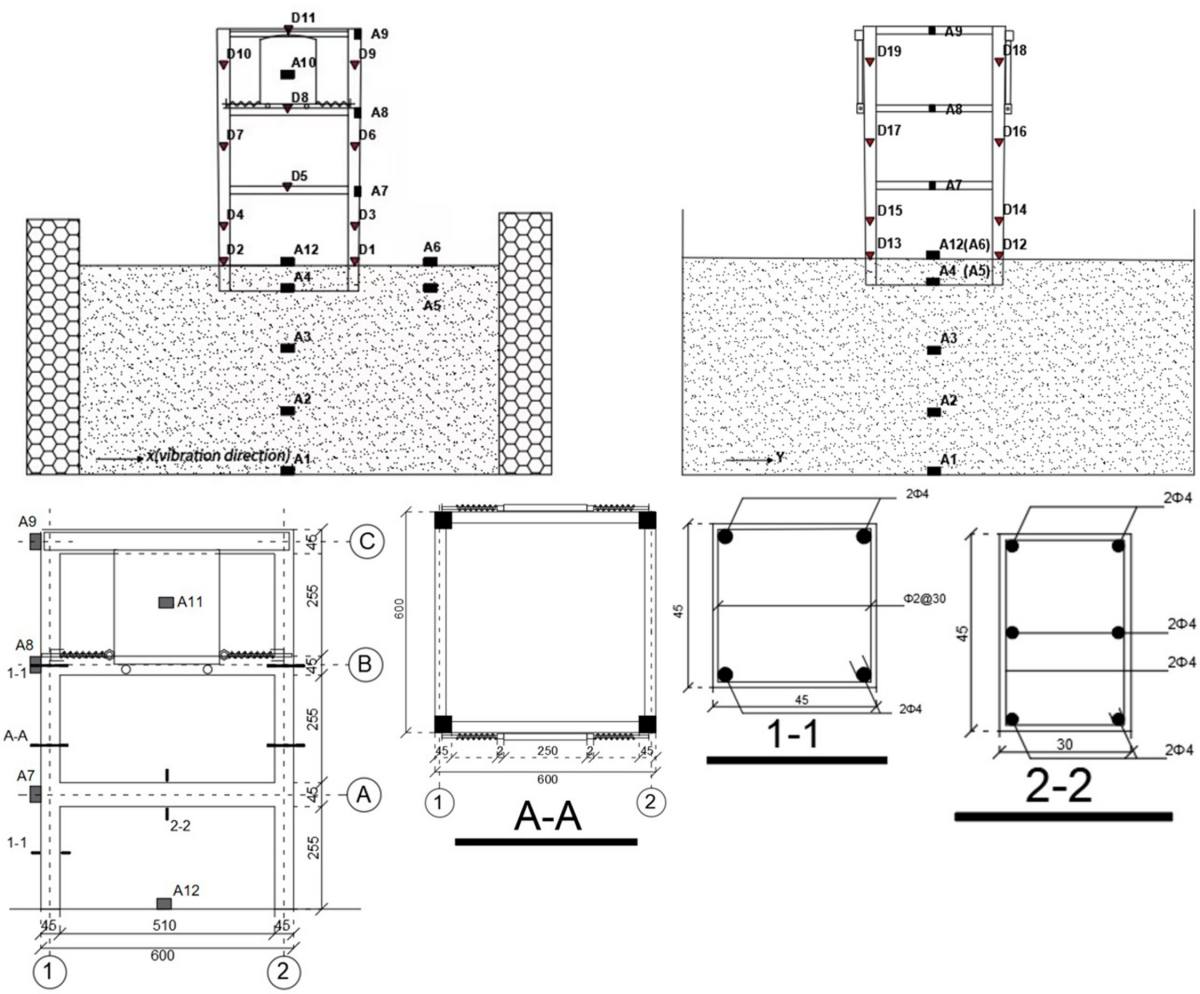
Measuring points layout.

In the soft soil foundation test model, acceleration sensors were arranged at six locations on the structure and soil. The six structurally arranged locations were located at the center of the foundation (A12), on the right side of each floor slab perpendicular to the vibration direction (A7, A8, and A9), and at the center of the wall-type TMD (A10 and A11). A11 is located on the wall-type TMD on the other side of the structure; therefore, it is not shown in Fig 3. The six locations arranged in the soil were at the center of the box bottom (A1), perpendicular to A1 (A2) at a depth of 300 mm, perpendicular to A1 (A3) at a depth of 600 mm, 500 mm to the right of A4 (A5), and perpendicular to the surface of A5 (A6). The strain sensors were located at the end of each column and edge of the floor slab, and the arrangements in the X- and Y-directions were the same. As shown in Fig 4.

## 3 Influences of wall damping TMD on the structure without SSI effects

The calculation formula of the TMD parameters derived from the rigid foundation in a previous study [24] was applied to the assembly wall-type damping TMD on a soft soil foundation, and shaking table tests were conducted. The seismic damping effect was verified by comparing the dynamic response of the structure under several different seismic excitations, acceleration peaks, and with and without wall-type damping TMD.

### 3.1 Experimental verification of boundary effect of model box

By collecting acceleration data from two acceleration sensors, A6 and A12, buried on the soil surface, the influence of boundary effects on the experimental testing was verified. The locations of the measurement points are shown in Fig 3. The measurement points A6 and A12 were located 500 mm apart. The model box effect in the experiment was analyzed by comparing the seismic characteristics measured at these two points.

**Fig 5:**
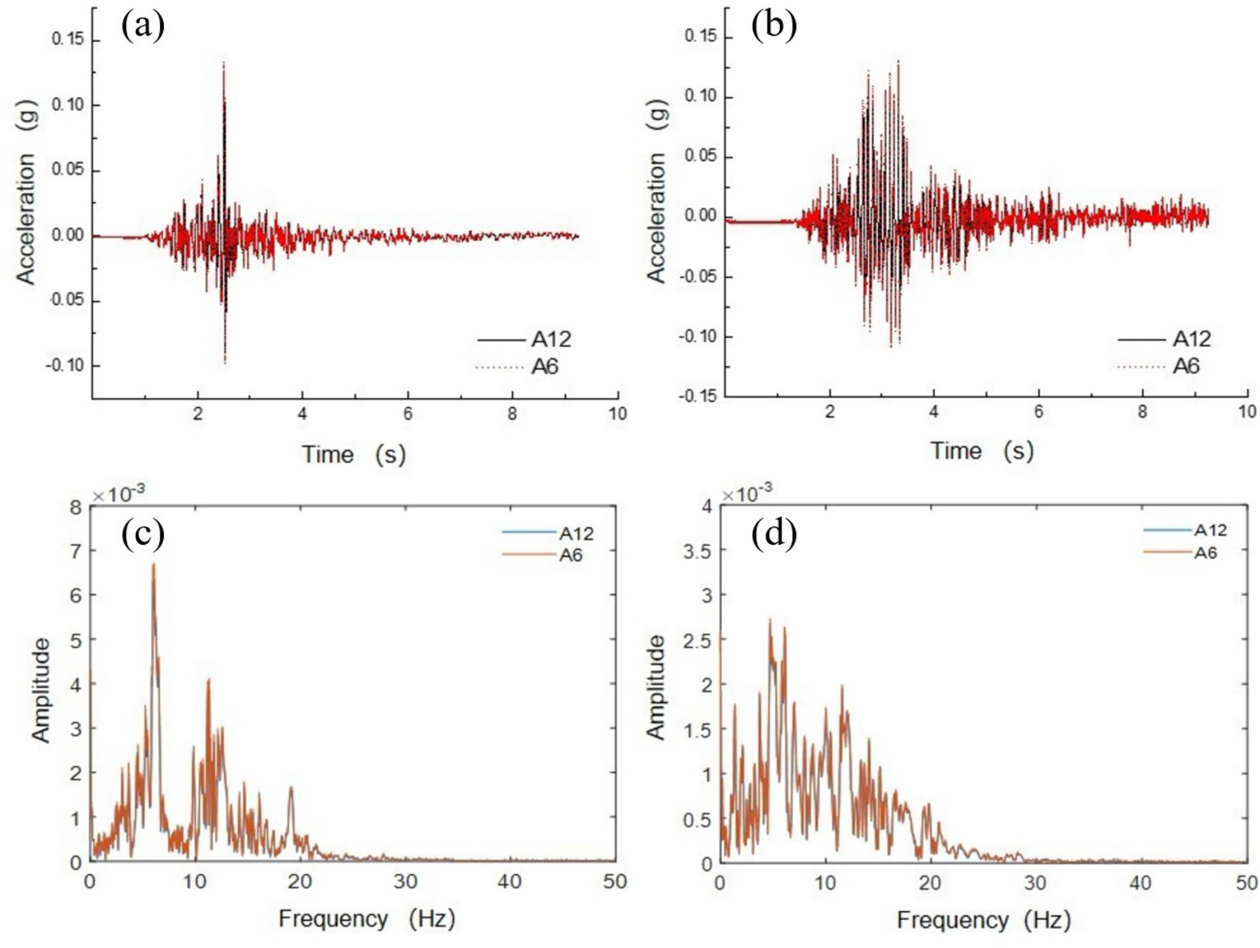
Comparison of acceleration–time histories and Fourier spectra between measuring points A6 and A12: (a) Acceleration–time curve for Kobe wave; (b) Acceleration–time curve for Taft wave; (c) Fourier spectrum for Kobe wave; (d) Fourier spectrum for Taft wave.

Using the peak acceleration as the intensity indicator of seismic motion, Fig 5(A) and 5(B) show the acceleration time history curves of measurement points A12 and A6, respectively, when inputting the Kobe and Taft waves. In the same cases, the acceleration time histories of the measurement points A12 and A6 were similar. This indicates that for point A6 near the boundary and point A12 farther away from the boundary, the influence of the boundary was insignificant.

Figs 5(C) and 5(D) show the Fourier spectrum curves comparison of measurement points A12 and A6. From the comparison curves, it can be observed that the results are similar to the acceleration time history comparison results, indicating that there are small differences in both the shape of the acceleration time history and the spectral components between measurement points A12 and A6.

Table 3 compares the data for measurement points A12 and A6 under different peak acceleration excitations. As the peak acceleration of the input seismic motion increased, the differences in the peak accelerations measured at points A12 and A6 also increased. However, these differences were relatively small. Therefore, the designed model box had slight impact on the peak acceleration.

**Table 3:** Comparison of different peak accelerations at measuring points A6 and A12.

Measuring point	Kobe wave (0.1 g)	Taft wave (0.1 g)	Kobe wave (0.3 g)	Taft wave (0.3 g)	Kobe wave (0.6 g)	Taft wave (0.6 g)
A12	0.121	0.118	0.322	0.355	0.628	0.584
A6	0.126	0.122	0.338	0.378	0.669	0.616
$|A_{6}-A_{12}|/A_{12}\times 100\text{\%}$	4.1	3.4	4.9	6.4	6.52	5.5

This indicates that the seismic response of measuring points A12 and A6 under this case is typically consistent. Specifically, the boundary of the model box had slight effect on the seismic response at measurement points A12 and A6, and the designed model box was reasonable.

### 3.2 Spectrum analysis

**Fig 6:**
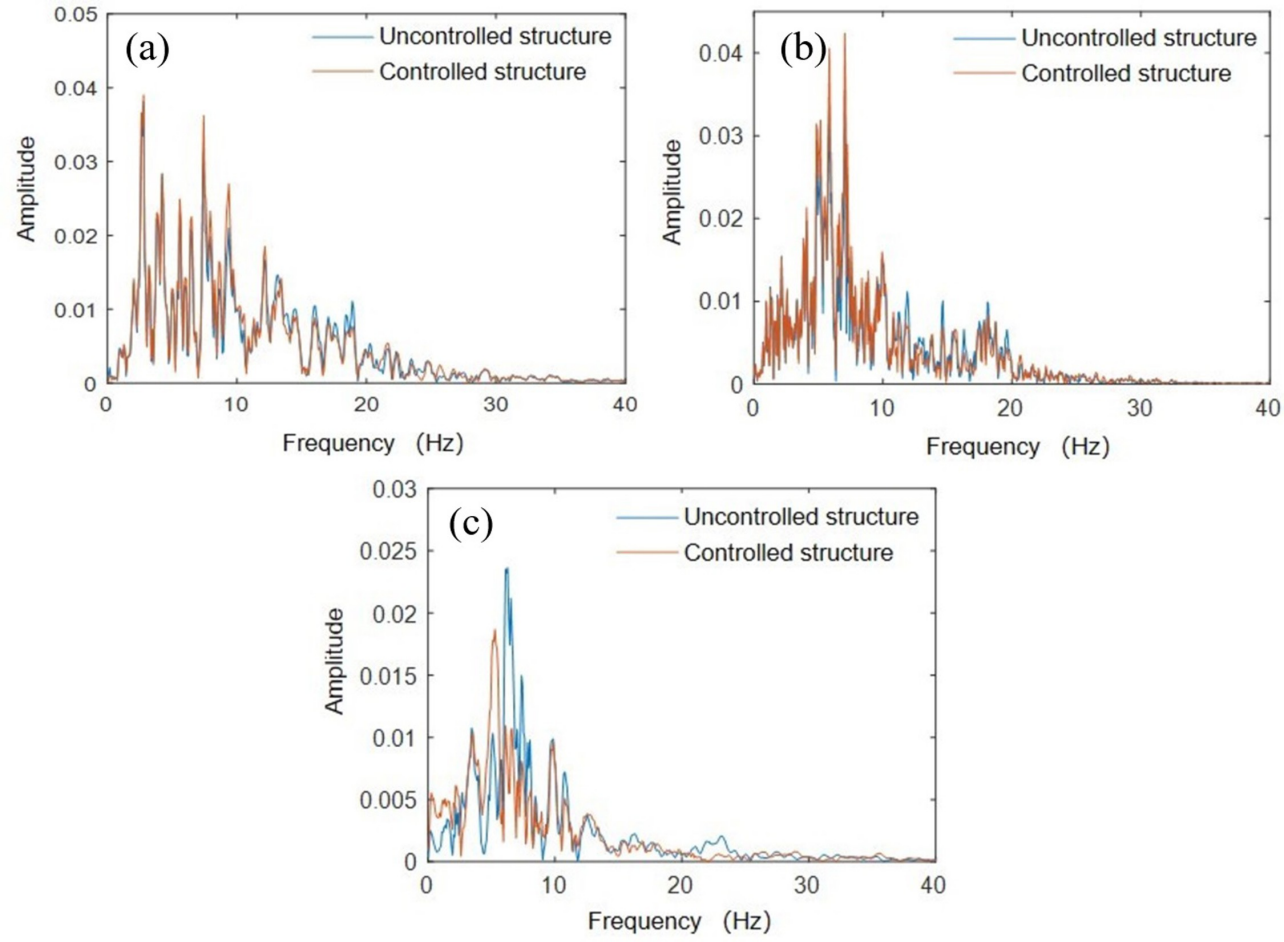
Fourier spectra of the top layer: (a) Kobe wave; (b) Taft wave; (c) Northridge wave.

A fast Fourier transform (FFT) was performed on the top layer acceleration collected from the soft soil foundation testing to understand the dynamic characteristic differences between the controlled and uncontrolled structures, as shown in Fig 6. From the figure, it can be observed that the peak Fourier transform under three different seismic excitations occurred near the natural vibration frequency of the structure at 6.96 Hz (6.74 Hz for structures with wall-type damping TMD installed, which is not affected by cumulative damage from the initial vibration test results), at 7.32 Hz, 7.07 Hz, and 6.22 Hz, respectively. The Fourier spectrum of the low-frequency component was amplified, and that of the high-frequency component was attenuated. For the controlled structure installed with the wall-type damping TMD, except for the Northridge wave, all other seismic waves had different degrees of amplification, indicating that the TMD parameters set by the traditional TMD based on the assumption of a rigid foundation may have an amplification effect on the soft foundation. For the controlled structures under the action of Northridge waves, the frequency of the Fourier peaks was lower than that of the uncontrolled structures. The reason for this phenomenon may be that the TMD parameters set on the rigid foundation are located in the effective frequency band of the Northridge waves.

### 3.3 Acceleration response analysis

**Fig 7:**
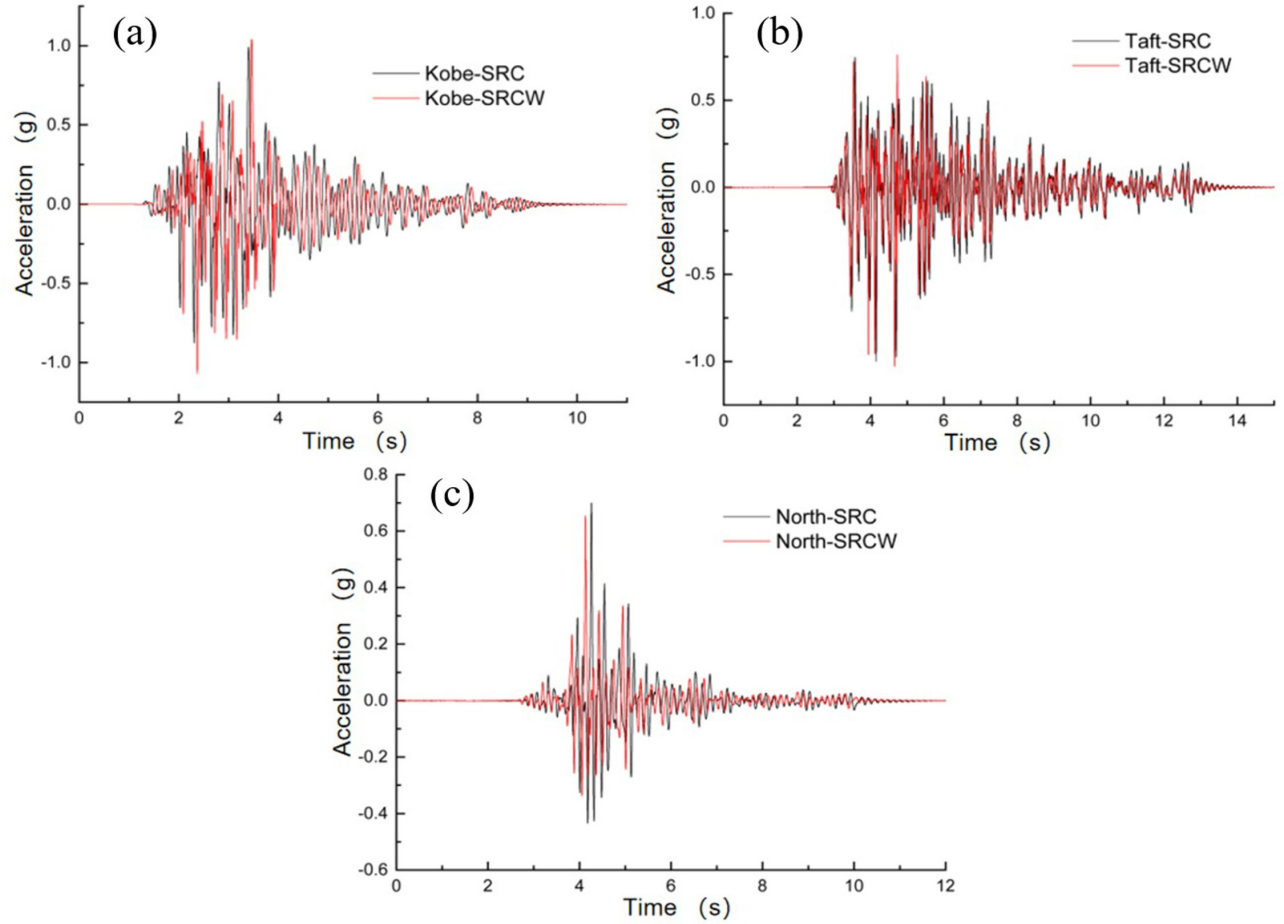
Acceleration–time histories of the top layer: (a) Kobe wave; (b) Taft wave; (c) Northridge wave.

To detect the seismic performance of traditional TMD that ignore SSI effects on soft soil foundations, shaking table tests were conducted on the frame model structure of a soft soil foundation under the action of Kobe, Taft, and Northridge waves. A comparison of the top-layer acceleration time histories for each case is shown in Fig 7. From the figure, it can be observed that regardless of whether it was a controlled structure with a wall-type damping TMD or an uncontrolled structure, the top layer acceleration time history curve was essentially consistent when the acceleration peak rose and fell, and the change trend was relatively similar. However, the wall-type damping TMD exhibited a vibration amplification effect on structural acceleration during certain seismic excitation processes. The maximum peak ground acceleration (PGA) response and damping rate of the top layer are listed in Table 4. Among them, under the action of Kobe wave, the average seismic reduction rate of wall-type damping TMD on structural floors was -2.62%, under the action of Taft wave, the average seismic reduction rate was -1.55%, and under the action of Northridge wave, the average seismic reduction rate was 4.37%.

**Table 4:** Comparison of acceleration damping rate.

Acceleration amplitude	Kobe wave			Taft wave			Northridge wave		
	Maximum value in uncontrolled state (g)	Maximum value of wall-type damping TMD (g)	Damping ratio (%)	Maximum value in uncontrolled state (g)	Maximum value of wall-type damping TMD (g)	Damping ratio (%)	Maximum value in uncontrolled state (g)	Maximum value of wall-type damping TMD (g)	Damping ratio (%)
0.1 g	0.198	0.201	-1.52	0.219	0.214	2.28	0.162	0.158	2.47
0.3 g	0.579	0.582	-0.52	0.590	0.586	0.68	0.380	0.364	4.21
0.5 g	0.752	0.779	-3.59	0.817	0.856	-4.77	0.642	0.613	4.52
0.6 g	0.991	1.039	-4.84	0.999	1.043	-4.40	0.699	0.655	6.29

**Fig 8:**
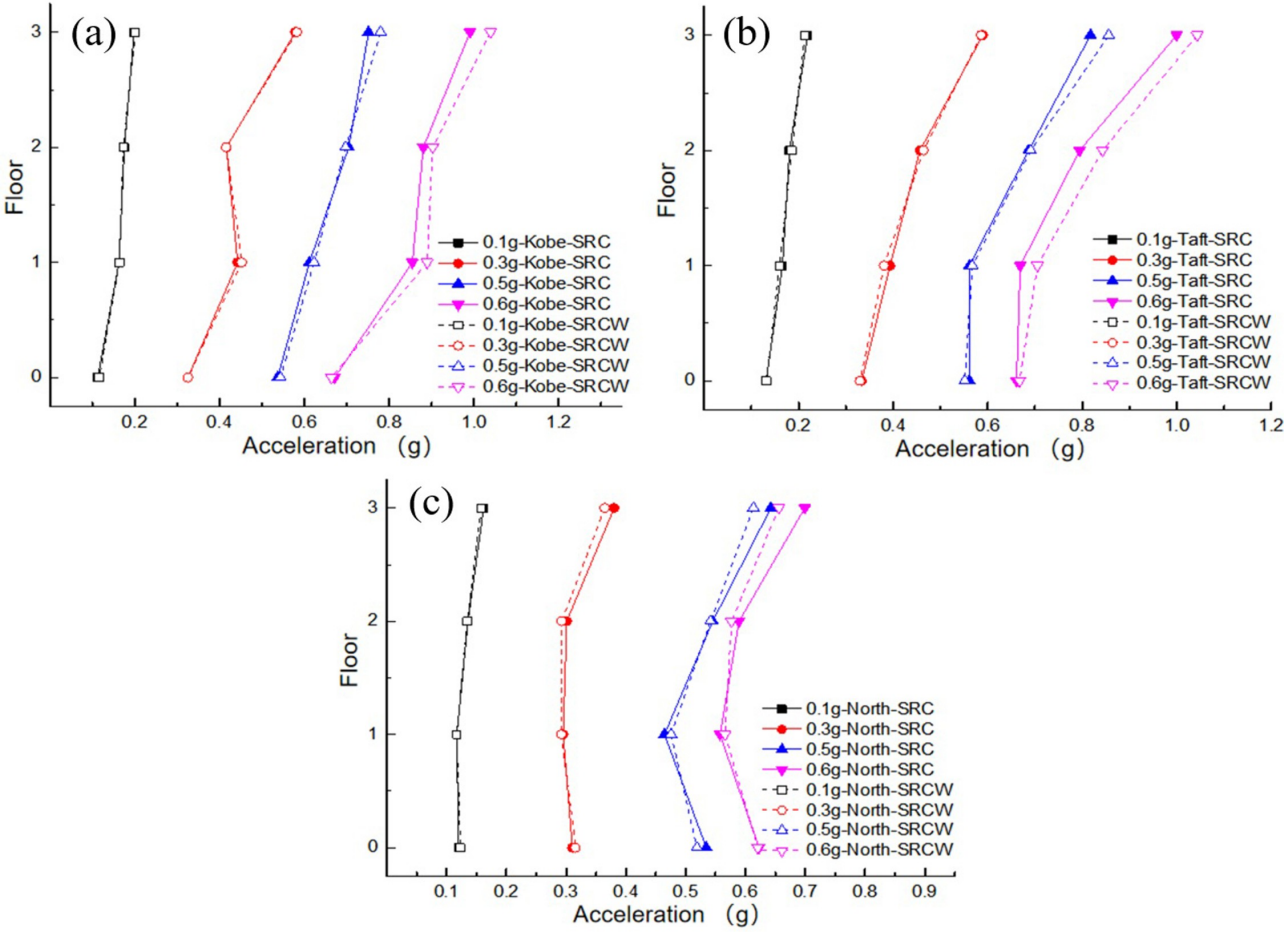
Comparison of peak acceleration of each floor: (a) Kobe wave; (b) Taft wave; (c) Northridge wave.

As the excitation peak increased, the acceleration of the structure under the soft soil foundation exhibited an amplification trend, similar to the response of the structure under a rigid foundation [24]. In addition, the spectral composition of the seismic waves had a significant influence on the damping effect of the wall-damping TMD; that is, the closer to the natural frequency of the structure, the greater the dynamic response of the structure. A comparison of the peak acceleration values for each floor under different peak acceleration excitations is shown in Fig 8. The figure shows that the acceleration response of the top layer of the structure was significantly greater than that of the bottom layer. The wall-type damping TMD, which ignored the SSI effect, exhibited a certain degree of acceleration response amplification under different seismic excitation peaks, except for the Northridge and Taft waves (0.1 g, 0.3 g), which achieved smaller seismic damping effects. This may be owing to the under high excitation conditions, the wall-type damping TMD could move and generate inertial forces. However, owing to the lack of an SSI effect in the parameter settings of the traditional TMD, there was a certain degree of detuning in the installed traditional TMD, which slightly increased the acceleration response of the structure.

### 3.4 Displacement response analysis

**Fig 9:**
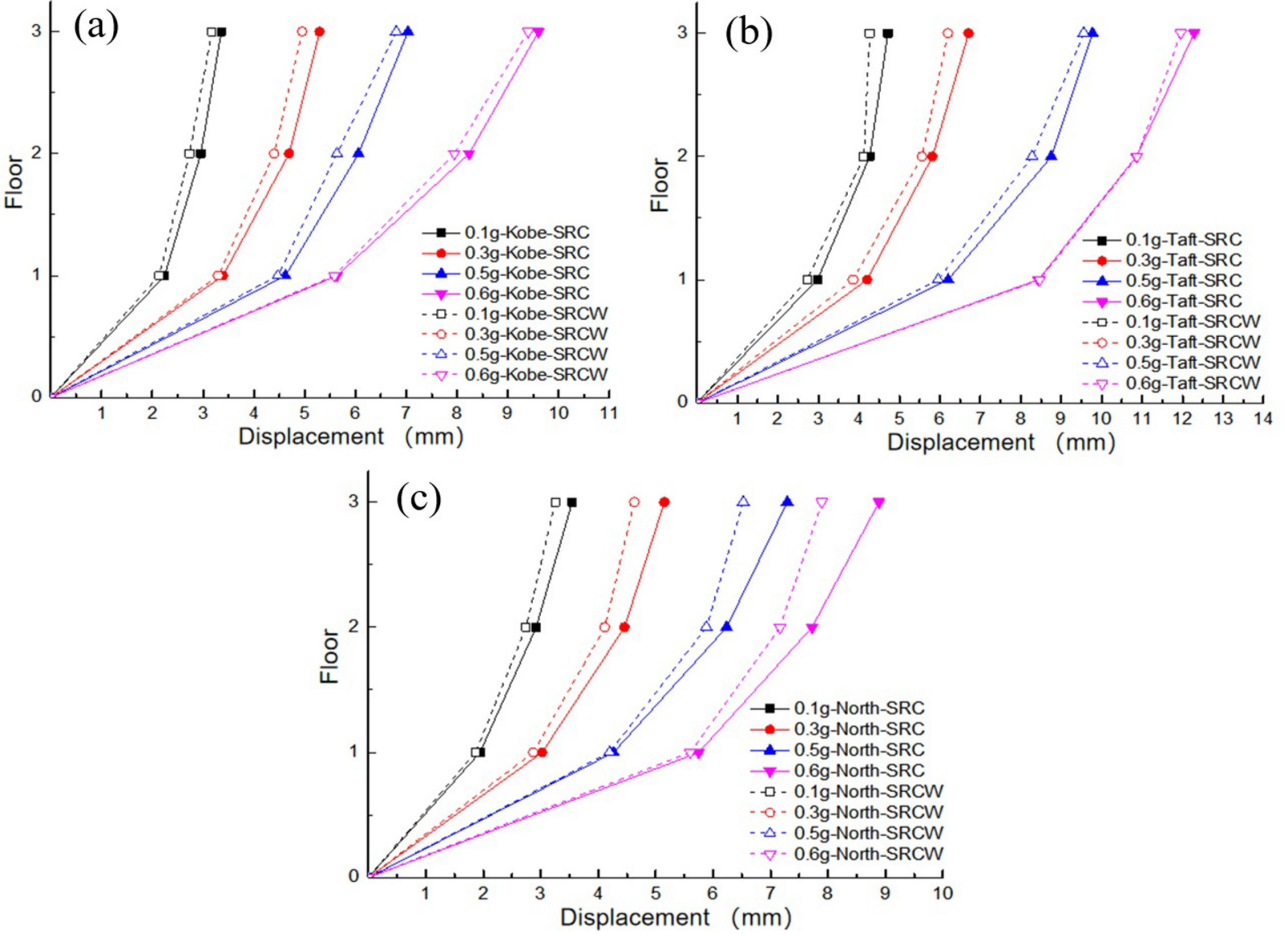
Comparison of peak displacement of each floor: (a) Kobe wave; (b) Taft wave; (c) Northridge wave.

In this experiment, acceleration data and displacement data were collected using a quadratic integration method to analyze the displacement response. To better detect the seismic performance of traditional TMD devices that ignore the SSI effects on soft soil foundations, the maximum displacement values of each layer of the controlled structure under the action of Kobe, Taft, and Northridge waves were compared. As shown in Fig 9, under the action of the three seismic waves, the maximum displacement of the top layer of the structure was greater than that of the bottom layer, which was similar to the acceleration response. The maximum displacement values under the Kobe and Taft waves were both greater than those under the Northridge wave. This may be owing to the predominant periods of Kobe and Taft waves are closer to the natural period of the structure, resulting in a more pronounced response compared to the action of Northridge wave. A comparison of the displacement damping rates for different excitation peaks is presented in Table 5. The average damping rates under the Kobe, Taft, and Northridge waves are 4.36%, 5.50%, and 10.05%, respectively. It can be observed that the displacement response of the structure slightly decreased after incorporating the traditional TMD device without considering the SSI effect. Under a high peak excitation acceleration, the wall-type damping TMD without considering the SSI effect exhibited poor seismic performance and did not significantly reduce the dynamic response of the structure. However, the wall-type damping TMD produced a small damping effect on the structural dynamics under the action of the Northridge wave, which may be owing to the large difference between the spectral components of the Northridge wave and those of the other two waves. The wall-damping TMD designed without considering SSI effects may be located in its effective frequency band.

**Table 5:** Comparison of displacement damping ratio.

Acceleration amplitude	Kobe wave			Taft wave			Northridge wave		
	Maximum value in uncontrolled state (mm)	Maximum value of wall-type damping TMD (mm)	Damping ratio (%)	Maximum value in uncontrolled state (mm)	Maximum value of wall-type damping TMD (mm)	Damping ratio (%)	Maximum value in uncontrolled state (mm)	Maximum value of wall-type damping TMD (mm)	Damping ratio (%)
0.1 g	3.36	3.17	5.65	4.72	4.27	9.53	3.55	3.25	8.45
0.3 g	5.29	4.95	6.43	6.71	6.20	7.60	5.15	4.63	10.10
0.5 g	7.03	6.80	3.27	9.78	9.56	2.25	7.29	6.53	10.43
0.6 g	9.60	9.40	2.08	12.28	11.96	2.61	8.89	7.89	11.25

## 4 Influences of SSI effects on the structure

For rigid foundation conditions, the structure is directly fixed on the shaking table surface; thus, the acceleration input of the shaking table surface is the seismic acceleration at the bottom of the structure; or soft soil foundation conditions, the soil box is fixed on the shaking table, and the acceleration record input from the shaking table reaches the bottom of the structure through the “filtering” of the soil layer. The acceleration measured by A12 was used as a comparison benchmark to analyze the impact of the SSI effects on the structure.

**Fig 10:**
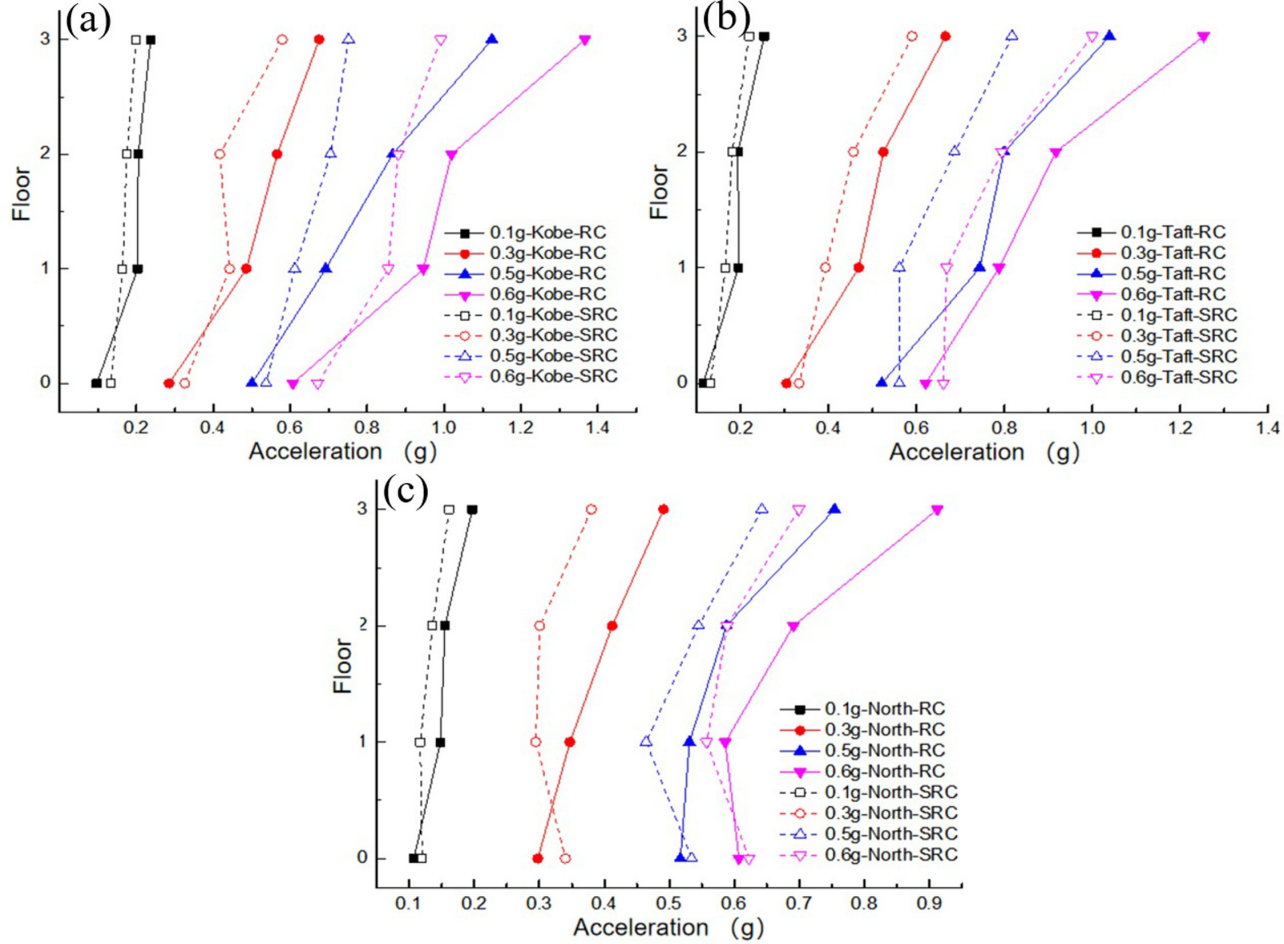
Comparison of peak acceleration of each floor: (a) Kobe wave; (b) Taft wave; (c) Northridge wave.

To analyze the influence of SSI effects on the structure, the dynamic response data measured under the RC case (rigid foundation without wall-type damping TMD frame structure) were compared with the data measured under the SRC case (soft soil foundation without wall-type damping TMD frame structure). A comparison of the accelerations of each layer of the structure under the action of the three seismic waves is shown in Fig 10. It can be observed from the figure that under the same peak acceleration input, there are differences in the response of structures under different types of seismic excitation. The Kobe and Taft waves were the largest, whereas the Northridge wave was the smallest. This may be because the spectral components of the former are close to the natural frequency of the system. In addition, it can be observed that there are certain differences in the peak acceleration magnitude and distribution form of floors under soft soil foundation conditions compared to those under rigid foundation conditions. With respect to the upper frame structure, the soil plays an obvious role in vibration isolation, which makes the vibration energy accepted by the upper structure small. The dynamic response of each floor is reduced to a certain extent compared with that of the rigid foundation, mainly because the soft soil foundation absorbs part of the external excitation energy. The maximum PGA responses and shock absorption rates for the different excitation peaks are listed in Table 6. It can be observed from the comparison results of the shock absorption rate that the shock absorption rate tended to increase with an increase in excitation.

**Table 6:** Comparison of acceleration damping rate.

Acceleration amplitude	Kobe wave			Taft wave			Northridge wave		
	Maximum value in uncontrolled state of rigid foundation (g)	Maximum value in uncontrolled state of soft soil foundation (g)	Damping ratio (%)	Maximum value in uncontrolled state of rigid foundation (g)	Maximum value in uncontrolled state of soft soil foundation (g)	Damping ratio (%)	Maximum value in uncontrolled state of rigid foundation (g)	Maximum value in uncontrolled state of soft soil foundation (g)	Damping ratio (%)
0.1 g	0.237	0.198	16.45	0.254	0.219	13.78	0.197	0.162	17.77
0.3 g	0.675	0.579	14.22	0.666	0.590	11.41	0.491	0.380	22.61
0.5 g	1.124	0.752	33.10	1.039	0.817	21.36	0.754	0.642	14.85
0.6 g	1.366	0.991	27.45	1.253	0.999	20.27	0.912	0.699	23.35

In summary, the SSI effect of the soil effectively reduced the dynamic response of the above ground structures, and the maximum reduction rate of the three seismic waves was 27%. However, the acceleration data collected on the soil surface were higher than the acceleration input from the shaking table. The reason for this phenomenon may be the movement of soil on the soil surface with the box body when the soil is excited, which is consistent with the conclusions of previous studies [19].

## 5 Shaking table tests of TMD on soft soil based on improved multi-population genetic algorithm

In this section, the motion equations of the building system considering soil-structure interaction (SSI) and tuned mass damper (TMD) interactions are established. A numerical model was developed to incorporate the SSI effects, and minimization of the transfer function peak was adopted as the objective function for the algorithm. The improved multipopulation genetic algorithm proposed in [25] was implemented using MATLAB to compute and optimize the TMD parameters. The optimized TMD was integrated into the structural model, and a subsequent shaking table test was conducted to validate its seismic performance.

### 5.1 Numerical model establishment for TMD-structure considering SSI effects

Owing to the coupling effect between the soil, structure, and TMD system, the consideration of SSI effects significantly affects the effectiveness of TMD vibration control. The optimal TMD parameters were consistent with the frequency of the structural system. Hence, the optimization results obtained without considering the SSI effects may not be optimal. Therefore, when addressing the placement of TMD devices in controlled structures, it is crucial to consider the influence of SSI.

In this study, a simplified calculation method was adopted to compute the SSI effects. The equations of motion for the entire system are as follows:

$$M^{\prime }\overset{`}{x}+C^{\prime }x+K^{\prime }x=-M_{g}I^{\prime }{\overset{`}{x}}_{g}$$ (1)

The mass, stiffness, and damping matrices of the soil-structure-TMD system in Eq (2) are as follows:

$$M^{\prime }=\left[\begin{array}{ccc} M & 0 & m_{i}\left(h_{i}+h_{0}\right)\\ 0 & m_{0} & m_{0}h_{0}\\ m_{i}\left(h_{i}+h_{0}\right) & m_{0}h_{0} & I_{f}+I_{0} \end{array}\right]$$

$$C^{\prime }=\left[\begin{array}{ccc} C & 0 & 0\\ 0 & c_{s} & 0\\ 0 & 0 & c_{r} \end{array}\right]$$

$$K^{\prime }=\left[\begin{array}{ccc} K & 0 & 0\\ 0 & k_{s} & 0\\ 0 & 0 & k_{r} \end{array}\right]$$

$$M_{g}=\left[\begin{array}{ccc} M & 0 & 0\\ 0 & \sum_{0}^{d}m_{i} & 0\\ 0 & 0 & \sum_{0}^{d}m_{i}h_{i} \end{array}\right]$$ (2)

Here, *M*′, *C*′ and *K*′ represent the mass, damping, and stiffness matrices, respectively, of the upper TMD structure system, which is of size *N* + 1 × *N* + 1; *I*′ is an *N* + 3 dimensional unit column vector; ${\overset{`}{x}}_{g}$denotes the input seismic acceleration; *h*_*i*_ represents the distances from each mass point in the upper structure to the foundation base; *I*_0_ is the moment of inertia of the upper TMD structure system about its own axis. The soil at the experimental site in this study was assumed to be homogeneous sand, indicating that there were only two sets of spring-damping systems. The energy-dissipation characteristics of the elastic elements and foundation can be calculated using Eqs (3) and (4). An equivalent foundation impedance independent of the frequency was adopted in this study. When the foundation has a certain burial depth, the horizontal spring stiffness coefficient *k*_*s*_ and rotational spring stiffness coefficient *k*_*r*_ are expressed by

$$k_{s}=\frac{8GR}{2-v}\left(1+2/3\frac{d}{R}\right)$$ (3)

$$k_{r}=\frac{8GR^{3}}{3(1-v)}\left(1+2\frac{d}{R}\right)$$ (4)

The damping coefficients *c*_*s*_ and *c*_*r*_ can be calculated using Eqs (5) and (6):

$$c_{s}=2.5AR$$ (5)

$$c_{r}=0.31AR^{2}$$ (6)

Here, *ρ* represents the soil density; *V*_*s*_ is the equivalent shear wave velocity of the soil; *v* denotes the Poisson’s ratio of the soil; *R* is the equivalent radius of the foundation. In the equation, *A* = *ρV*_*s*_*R*.

### 5.2 Selection of the objective function

In the optimization of the TMD parameters, one can either minimize the peak acceleration as the objective function [33] or consider both the acceleration and displacement of the controlled structure, integrating them into a problem to obtain Pareto optimal solutions. However, this type of TMD optimization is highly dependent on the input seismic waves because each earthquake event is stochastic, rendering the sought TMD optimization non-generalizable. The transfer function can significantly reflect the variation in the structural dynamic amplification factor with excitation frequency under seismic excitations. To maintain generality, the minimization of the peak value of the transfer function was selected as the optimization objective [14, 34]. The expression for the transfer function remains consistent with that proposed in the previous section [16], with matrices *M*, *C* and *K* taken from Section 5.1 as matrices *M*′, *C*′ and *K*′, respectively.

To obtain more accurate parameters for the wall-type tuned-mass damper (TMD), an improved multipopulation genetic algorithm was utilized for 100 iterations. The optimization aims to minimize the peak value of the transfer function for the upper structure. The objective function is defined as

$$G(X)=min\left(\left(H_{x}(\omega )\right)\max\right)$$ (7)

The final TMD parameters obtained by incorporating the dynamic soil parameters into the model and performing optimization are listed in Table 7.

**Table 7:** Structural optimization results.

Mass Ratio *μ*	Mass (*kg*)	Period (*rad*/*s*)	Stiffness (*N*/*mm*)	Damping Coefficient (*Ns*/*m*)
0.06	7	35.01	8.22	64.0

**Fig 11:**
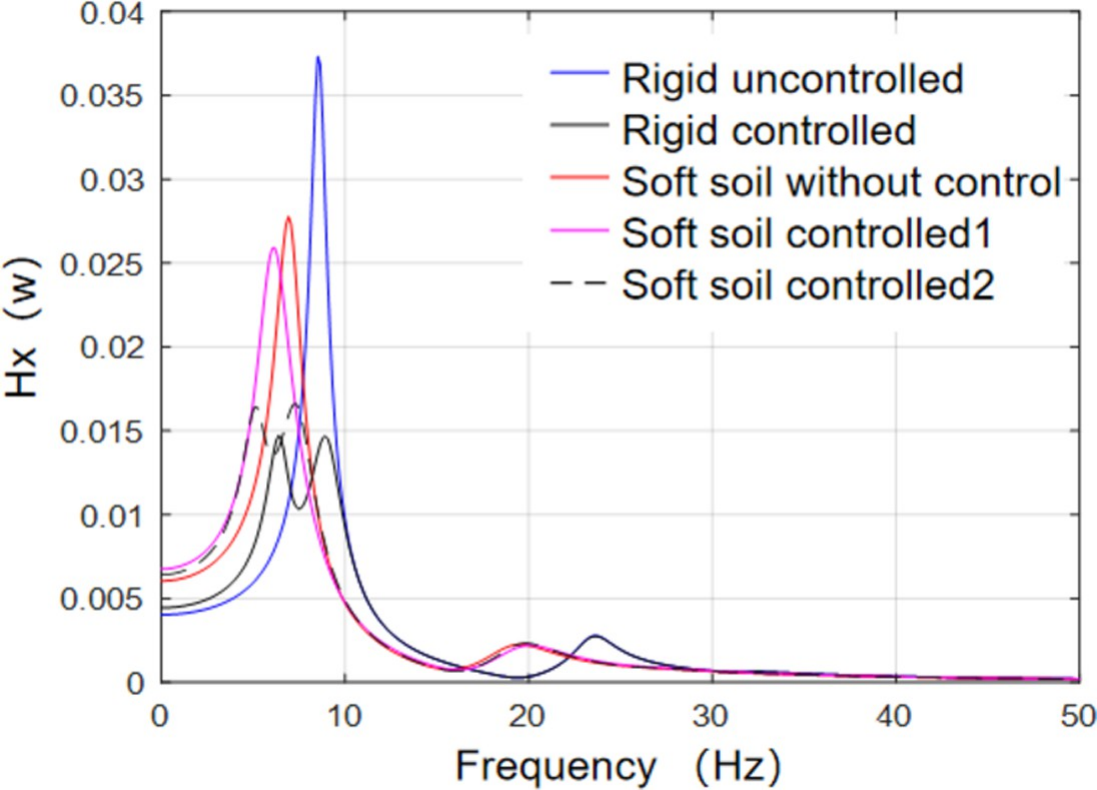
Comparison of transfer functions.

According to the above transfer function calculation formula, with a frequency step of 0.2, the transfer function graphs for different foundation conditions were computed and plotted in MATLAB, as shown in Fig 11. “Soft soil controlled 1” refers to the wall-type tuned mass damper (TMD) controlled structure with rigid foundation conditions, while “Soft soil controlled 2” refers to the wall-type TMD controlled structure with optimized parameters considering soft soil foundation conditions. It can be observed that for the structure with a rigid foundation, the designed wall-type TMD significantly reduces the peak value of the transfer function. However, the reduction capabilities of structures with soft soil foundations are limited. Nevertheless, the transfer function peak for the soft soil foundation was smaller than that for the rigid foundation, and the extent of the reduction depended on the parameters of the underlying soil. Furthermore, a comparison reveals that the wall-type TMD, considering the SSI effects and optimized based on the soil-structure numerical model, shows a noticeable reduction in the transfer function peak compared to the uncontrolled structure on a soft soil foundation. However, the damping effect was not as significant as that on a rigid foundation, which is consistent with the conclusions drawn in references [7, 11] based on experimental and numerical simulations. Subsequent shaking-table tests were performed to further verify this damping effect.

### 5.3 Acceleration response analysis

**Fig 12:**
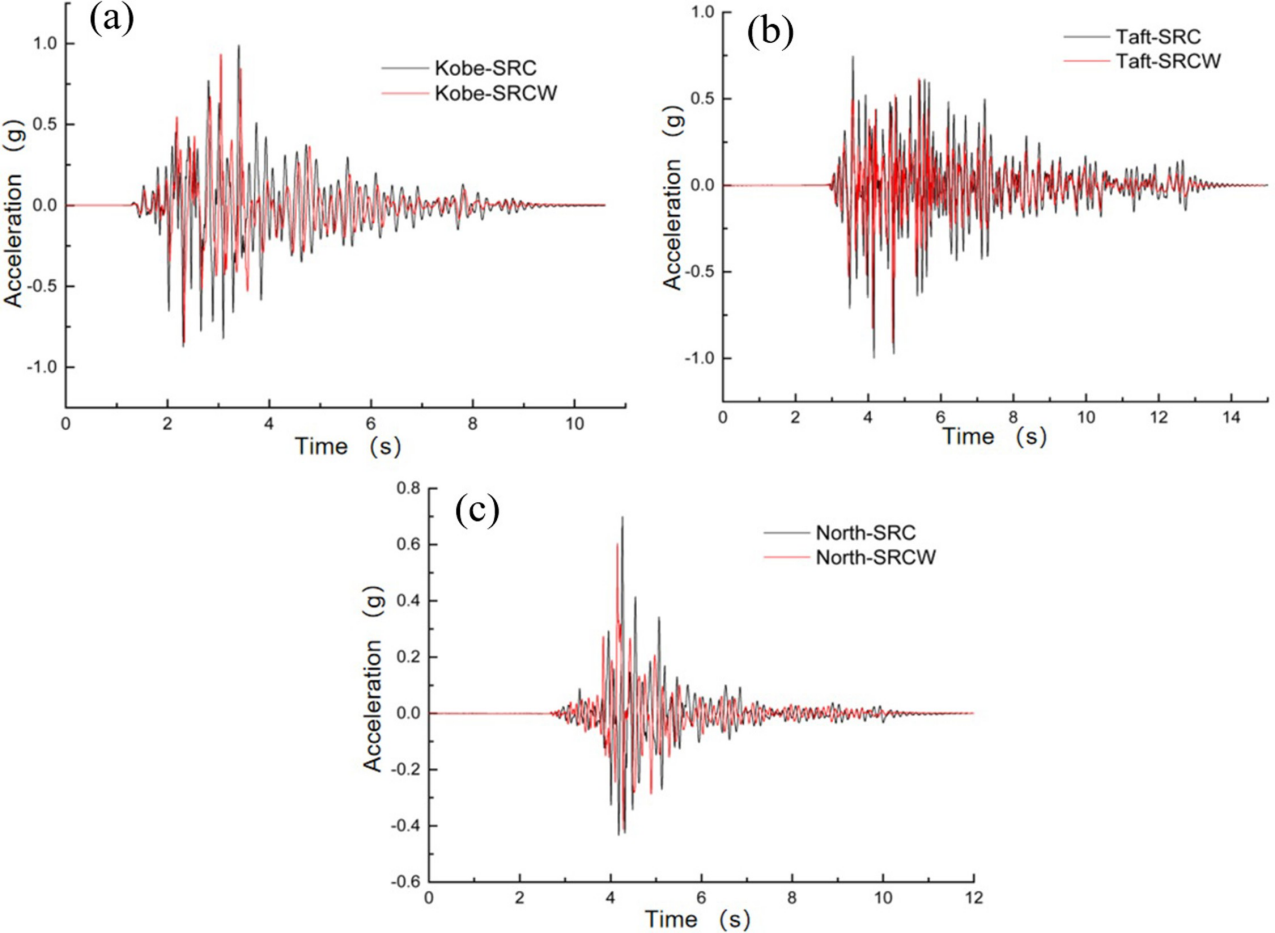
Acceleration–time histories of the top layer: (a) Kobe wave; (b) Taft wave; (c) Northridge wave.

To evaluate the seismic performance of the wall-type tuned mass damper (TMD) considering the SSI effects, a shaking table test was conducted on the frame model structure on a soft soil foundation following the methodology described in Section 3. The time history of the top-floor acceleration response is shown in Fig 12. It is evident from the figure that the acceleration response of the controlled structure is reduced to varying degrees throughout the entire process, with a more pronounced reduction in the peak acceleration. The peak accelerations and damping ratios are listed in Table 8. The average damping ratios under the influence of Kobe, Taft, and Northridge waves were 8.18%, 12.05%, and 10.29%, respectively. By analyzing the dynamic response characteristics of the controlled structure, it was observed that as the PGA increased, the acceleration of the structure tended to increase. In addition, the spectral components of the seismic wave significantly influenced the damping performance of wall-type TMD. Generally, when the seismic wave frequency approaches the natural structural frequency, the structural dynamic response increases.

**Table 8:** Comparison of acceleration damping rate.

Acceleration amplitude	Kobe wave			Taft wave			Northridge wave		
	Maximum value in uncontrolled state (g)	Maximum value of wall-type damping TMD (g)	Damping ratio (%)	Maximum value in uncontrolled state (g)	Maximum value of wall-type damping TMD (g)	Damping ratio (%)	Maximum value in uncontrolled state (g)	Maximum value of wall-type damping TMD (g)	Damping ratio (%)
0.1 g	0.198	0.187	5.55	0.219	0.198	9.59	0.162	0.151	6.79
0.3 g	0.579	0.529	8.64	0.590	0.503	14.75	0.380	0.346	8.95
0.5 g	0.752	0.675	10.24	0.817	0.717	12.24	0.642	0.565	11.99
0.6 g	0.991	0.909	8.27	0.999	0.883	11.61	0.699	0.605	13.45

**Fig 13:**
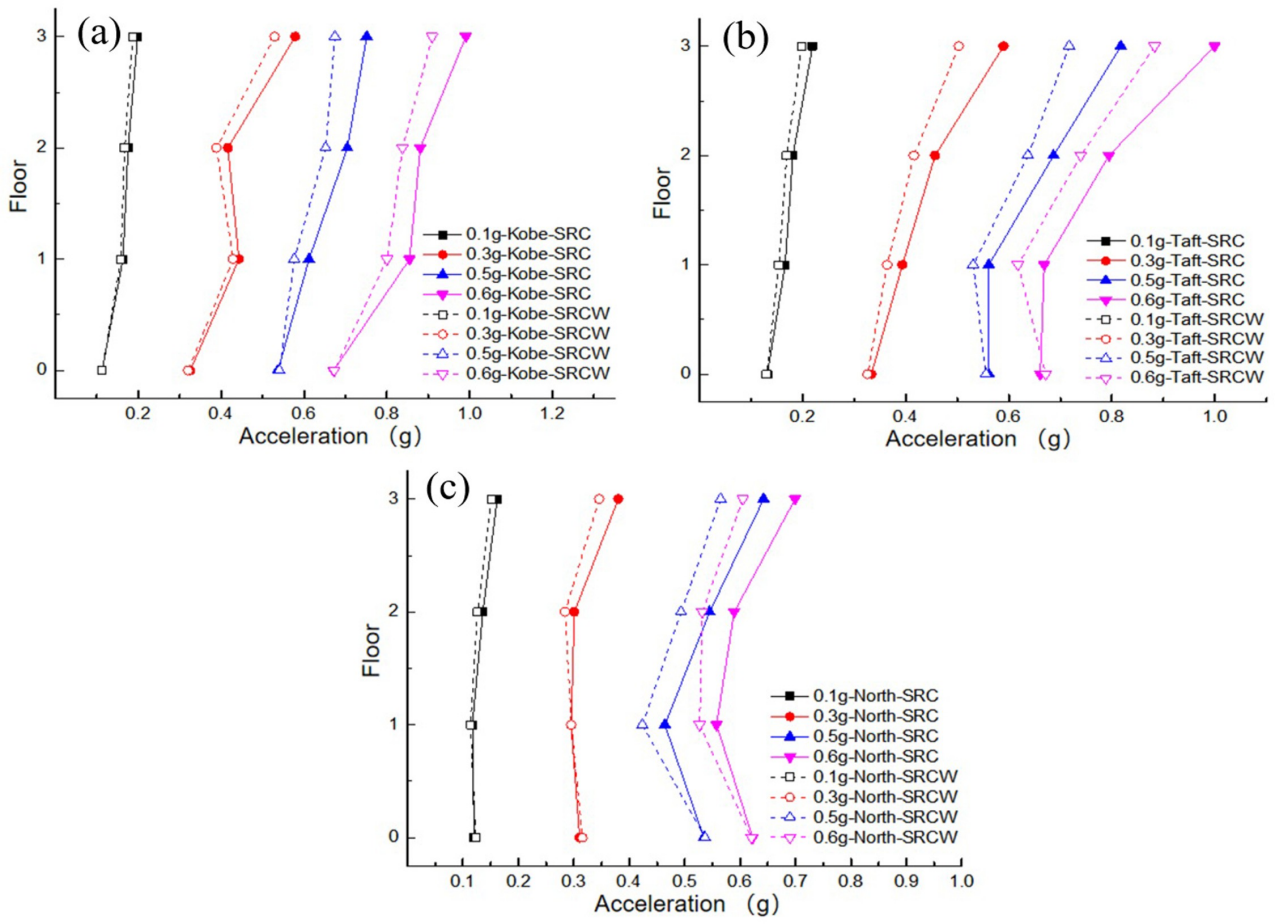
Comparison of peak acceleration of each floor: (a) Kobe wave; (b) Taft wave; (c) Northridge wave.

A comparison of peak accelerations for each floor under different PGAs is shown in Fig 13. The acceleration response on the top floor of the structure was significantly higher than that on the bottom floor. A wall-type tuned mass damper (TMD) exhibits a certain damping effect on each floor under various seismic excitations. Notably, the damping effect was more pronounced on the top floor, which was likely owing to the placement of the wall-type TMD system at the top of the structure. The system experiences motion and generates opposing inertial forces that act directly on the top floor, which partially offsets the excitation energy. Consequently, the damping effect was more significant on the top floor than on the lower floors, and the damping ratio was proportional to the peak external excitation, which is similar to the behavior observed on rigid foundations. However, Table 8 shows that when the external excitation reaches 0.6 g, the damping ratio decreases compared to the case at 0.5 g. This may be attributed to the nonlinear behavior of the structural materials, which causes a certain degree of mismatch in the designed wall-type TMD parameters, thereby affecting the damping performance.

### 5.4 Displacement response analysis

**Fig 14:**
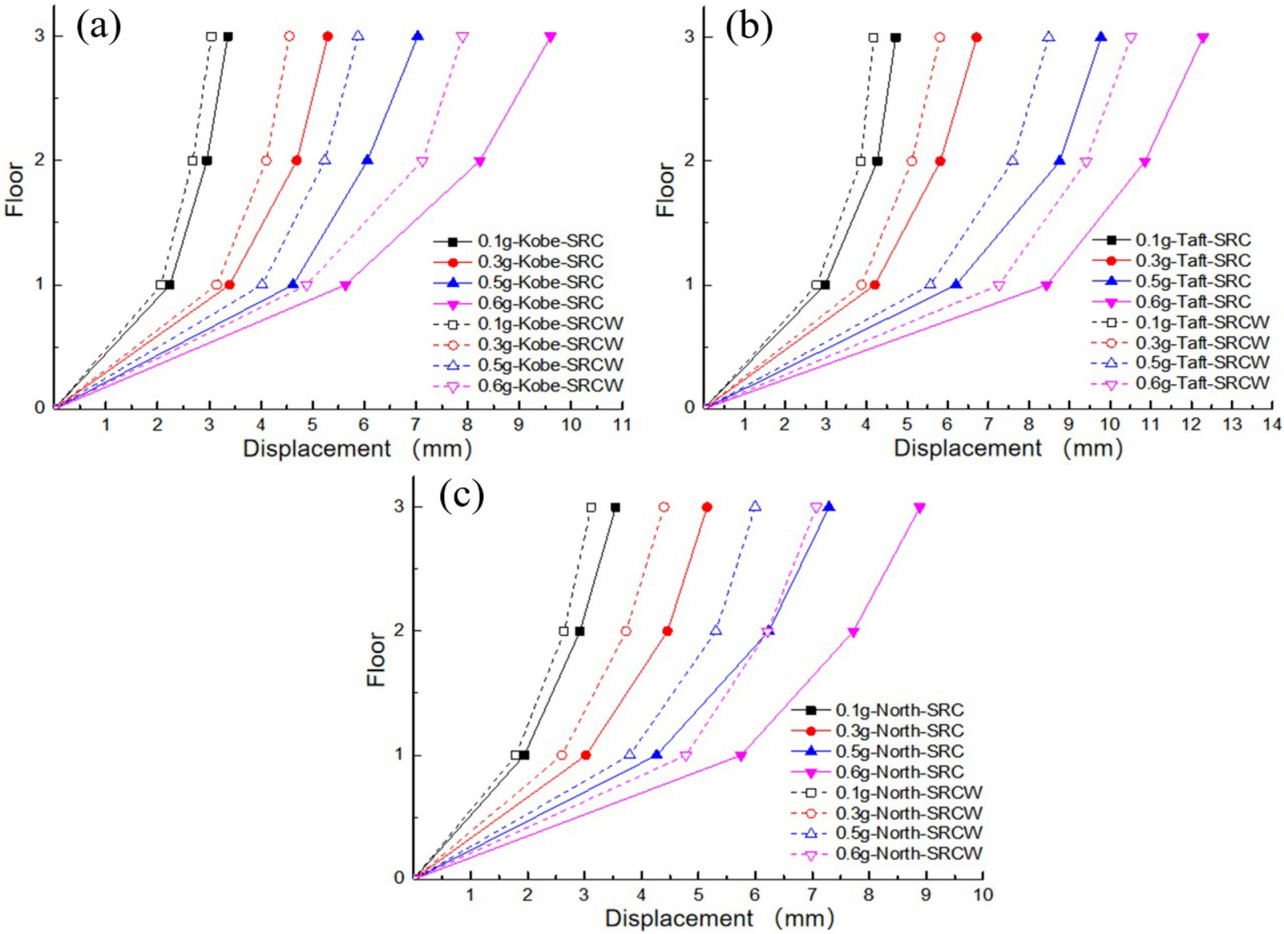
Comparison of peak displacement of each floor: (a) Kobe wave; (b) Taft wave; (c) Northridge wave.

To further analyze the seismic performance of the wall-type tuned mass damper (TMD) considering the SSI effects on a soft soil foundation, a comparison of the maximum displacements at each floor of the controlled structure was conducted. From Fig 14, it can be observed that under the influence of the three seismic waves, the trend of the displacement variation at the top floor of the structure is consistent with that in Section 3.4, which is related to the dominant period of the seismic waves and the natural period of the structure. A comparison of the maximum damping ratios for each case is presented in Table 9, where the average damping ratios under the influence of the Kobe, Taft, and Northridge waves are 14.395%, 13.11%, and 16.27%, respectively.

**Table 9:** Comparison of displacement damping ratio.

Acceleration amplitude	Kobe wave			Taft wave			Northridge wave		
	Maximum value in uncontrolled state (mm)	Maximum value of wall-type damping TMD (mm)	Damping ratio (%)	Maximum value in uncontrolled state (mm)	Maximum value of wall-type damping TMD (mm)	Damping ratio (%)	Maximum value in uncontrolled state (mm)	Maximum value of wall-type damping TMD (mm)	Damping ratio (%)
0.1 g	3.36	3.04	9.52	4.72	4.18	11.44	3.55	3.11	12.39
0.3 g	5.29	4.55	13.99	6.71	5.81	13.41	5.15	4.40	14.56
0.5 g	7.03	5.88	16.36	9.78	8.49	13.19	7.29	6.00	17.69
0.6 g	9.60	7.90	17.71	12.28	10.51	14.41	8.89	7.07	20.47

As observed, the addition of a wall-type tuned-mass damper (TMD) effectively reduces the displacement response of the structure, and the reduction effect becomes more pronounced with an increase in the peak ground acceleration. Tuned-mass dampers primarily control the first mode of the structure. Hence, placing the wall-type TMD at the top of the structure resulted in a more significant displacement control effect on the top floor than on the lower floors. Comparing the damping effects on the displacement and acceleration, the wall-type TMD exhibited better displacement control than acceleration control.

## 6 Conclusions

In this study, shaking table tests were conducted on an assembled wall-type tuned mass damper (TMD) with and without considering the soil-structure interaction (SSI) effects on a soft soil foundation. Using the improved multipopulation genetic algorithm in MATLAB, the TMD parameters considering SSI effects were optimized based on the minimization of the transfer function peak value. The optimized TMD parameters were integrated into the structural model to verify the seismic performance of the wall-type TMD with and without considering SSI effects. A comparison was made with the dynamic response of the uncontrolled structure on both rigid and soft soil foundations to analyze the influence of the SSI effects on the structure. The main conclusions are as follows.

1. Under soft soil foundation conditions, traditional TMD design parameters, without considering the SSI effects, led to a certain degree of mismatch, resulting in a limited reduction in the dynamic response and even dynamic amplification in some seismic excitations.
2. The study of the SSI effects revealed that soil plays a significant isolating role, leading to reduced vibration energy being transmitted to the upper structure. Compared with the rigid foundation, all floors showed some degree of reduction in the dynamic response on a soft soil foundation, and the damping ratio increased with increasing excitations.
3. Considering the SSI effects and optimizing the wall-type TMD parameters using the improved multipopulation genetic algorithm led to a significant reduction in the dynamic response of the controlled structure on the soft soil foundation. Both acceleration and displacement showed increased reduction effects with increasing excitation, and the damping ratios for acceleration and displacement were proportional to the peak external excitation, with maximum reductions of 13.45% and 20.47%, respectively.

Therefore, the optimization of TMD parameters using the improved multi-population genetic algorithm in this study provides valuable insights into the design and application of tuned mass dampers considering soil-structure interaction (SSI) effects. These findings have engineering significance for improving the seismic performance of structures. In future research, various building information modeling (BIM) big data techniques could be utilized to account for different soil properties under various foundation conditions. Neural network algorithms can be trained to predict the impact of the TMD parameters on a structure, thereby enabling the selection of suitable TMD parameters for specific scenarios. This approach further enhances the efficiency and accuracy of TMD designs and practical engineering applications.

## Supporting information

S1 Data (ZIP)
